# A novel semi-flexible coaxial nozzle based on fluid dynamics effects and its self-centering performance study

**DOI:** 10.1038/s41598-024-66623-8

**Published:** 2024-07-06

**Authors:** Yu Li, Shilei Li, Xiaobo Du, Haijun Qu, Jianping Wang, Pingyan Bian, Haiguang Zhang, Shuisheng Chen

**Affiliations:** 1 School of Mechanical and Power Engineering, Henan Polytechnic University, Jiaozuo, Henan China; 2 National Demonstration Center for Experimental Engineering Training Education, Shanghai University, Shanghai, China

**Keywords:** Coaxial nozzle, Semi-flexible, Coaxiality, Fluid–solid coupling, Self-centering, Mechanical engineering, Gels and hydrogels

## Abstract

Coaxial nozzles are widely used to produce fibers with core–shell structures. However, conventional coaxial nozzles cannot adjust the coaxiality of the inner and outer needles in real-time during the fiber production process, resulting in uneven fiber wall thickness and poor quality. Therefore, we proposed an innovative semi-flexible coaxial nozzle with a dynamic self-centering function. This new design addresses the challenge of ensuring the coaxiality of the inner and outer needles of the coaxial nozzle. First, based on the principles of fluid dynamics and fluid–structure interaction, a self-centering model for a coaxial nozzle is established. Second, the influence of external fluid velocity and inner needle elastic modulus on the centering time and coaxiality error is analyzed by finite element simulation. Finally, the self-centering performance of the coaxial nozzle is verified by observing the coaxial extrusion process online and measuring the wall thickness of the formed hollow fiber. The results showed that the coaxiality error increased with the increase of Young’s modulus E and decreased with the increase of flow velocity. The centering time required for the inner needle to achieve force balance decreases with the increase of Young's modulus ($E$) and fluid velocity (${v}_{f}$). The nozzle exhibits significant self-centering performance, dynamically reducing the initial coaxiality error from 380 to 60 μm within 26 s. Additionally, it can mitigate the coaxiality error caused by manufacturing and assembly precision, effectively controlling them within 8 μm. Our research provides valuable references and solutions for addressing issues such as uneven fiber wall thickness caused by coaxiality errors.

## Introduction

Coaxial extrusion technology is a method that deposits two or more materials simultaneously along the same longitudinal axis to manufacture capsules or fiber materials with core–shell or multi-core–shell structures^1,2^. Coaxial extrusion technology has demonstrated significant value and broad application prospects in various fields such as bio-scaffolds^3^, vascular networks^4–6^, drug delivery^7^, sensors^8–10^, carbon-based conductive nanomaterials^11^, electronic textiles^12^, flexible batteries^13^, laser cladding^14,15^, and more.

Sang-Hoon Lee^16^ used a coaxial microfluidic nozzle to react and form alginate fibers. This method, which can be continuously extruded and cured at room temperature, has many advantages, such as controllable fiber size, a simple process, and convenient loading of cells or growth factors. Mistry et al.^17^ utilized cell-laden hydrogels as the core material, partially crosslinked alginate or hybrid hydrogels composed of alginate and polyethylene glycol diacrylate (PEGDA) as the shell material to fabricate cell-laden fibrous vessels with a diameter of approximately 800 µm. Morteza Bazgir et al.^18^ prepared degradable porous scaffolds through the coaxial extrusion of poly(ε-caprolactone) (PCL) and Poly (lactic-co-glycolic acid) (PLGA). This nanofiber scaffold exhibits excellent mechanical properties and enhances tissue engineering scaffolds' performance. The above studies realized the direct extrusion forming of a solid core or hollow fiber of alginate hydrogel, which laid a foundation for the direct writing of alginate gel. In particular, the realization of the hollow fiber structure promoted the integration of the vascular function and scaffold structure. Giovanni Falcone^19^ coaxially extruded sodium alginate solution (shell) and crosslinking agent (core, hydroxyethyl cellulose 3%, calcium chloride 0.1 M, and propranolol hydrochloride 12.5% w/v) to print the concentric geometry of the ring model. The technology could realize the personalized drug dosage formulation according to the patient's treatment plan. Furthermore, Li yun Huang^20^ utilized three cylindrical tubes (injection, transition, and collection tubes) to seal and assemble a microfluidic device fixed on a glass plate with epoxy resin. This device prepares liquid droplets or microcapsules and finds applications in drug delivery, controlled release, cell encapsulation, and other fields. Zhejiang University's Yong He et al.^21^ employed coaxial extrusion of silicone gel and liquid metal; the coaxial pipe coil would be printed on the cylinder, formed containing a liquid metal hollow spiral pipe. And then, it made a multifunctional inductance flexible sensor by 3D printing. This hollow cylindrical structure made the sensor fit perfectly with the shape of a soft serpentine robot. Qi Wang et al.^22^ produced highly flexible fiber electrodes through coaxial extrusion of MnO_2_-based active electrodes and carboxymethyl cellulose (CMC) spinning solution. Subsequently, these fibers were cut into segments, immersed in LiCl-PVA gel electrolyte, coated, and reassembled to obtain fiber supercapacitors. Hui Wu et al.^23^, through the coaxial extrusion of poly (APhe-co-AAm) and PVDF-HFP&PU, prepared composite fibers with outstanding stretchability and conductivity, suitable for fabricating flexible wearable electronic devices.

Although coaxial extrusion technology has been successfully applied in various fields, the coaxial precision of the coaxial nozzle still needs improvement. Without this precision, the uniformity of the core–shell structure of the corresponding products cannot be guaranteed, potentially affecting their functionality. Many scholars have conducted extensive research on the structure and coaxiality error of the coaxial nozzle.

**Figure 1:**
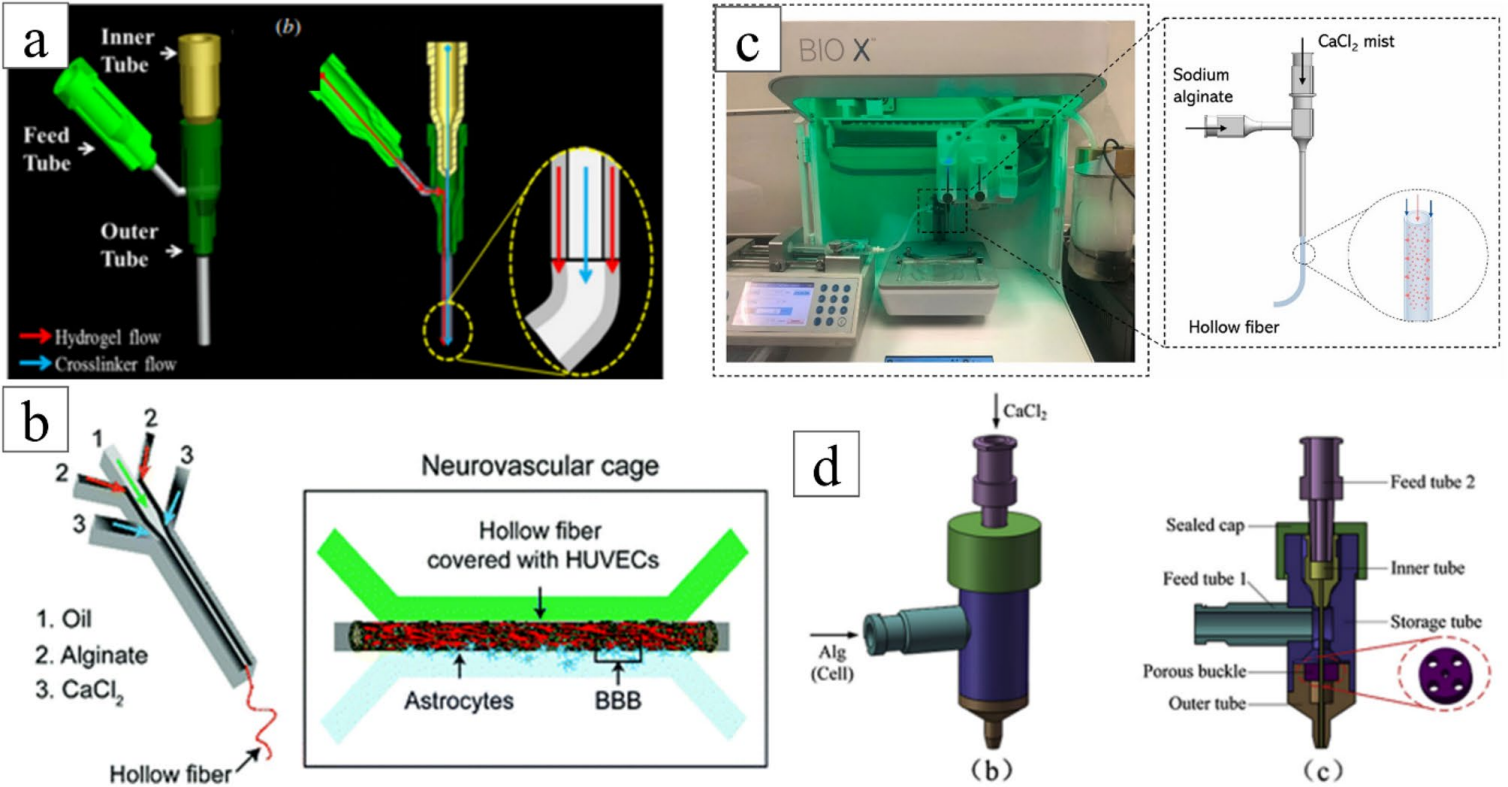
Coaxial nozzle structure (**a**) 3D model of a coaxial nozzle (Reproduced with permission of Ref.^24^. Copyright 2013, IOP Publishing). (**b**) Triple-flow PDMS microfluidic device (Reproduced with permission of Ref.^4^. Copyright 2018, Royal Society of Chemistry). (**c**) Coaxial nozzle with added atomizer (Reproduced with permission of Ref.^26^. Copyright 2023, Elsevier B.V). (**d**) Using porous latches to control the coaxiality of the coaxial nozzle (Reproduced with permission of Ref.^28^. Copyright 2015, Elsevier B.V).

Ibrahim T Ozbolat et al.^24^ of the University of Iowa directly combined three dispensing needles and glued them to secure the seal, as shown in Fig. 1a. Buu Minh Tran^4^ fabricated a triple-flow PDMS microfluidic device through mold manufacturing. It consists of three coaxial channels (inner, middle, and outer channels) and one outlet, as shown in Fig. 1b. Peng Ju Wang et al.^25^ utilized commercial 3D printers to manufacture molds and then performed precision assembly on these molds. The above two devices processed through molds can effectively ensure the coaxiality of the coaxial nozzle. However, this device's manufacturing process is complex, expensive, and requires high assembly precision. Sara Badr^26^ installed an atomizer on the coaxial nozzle to atomize calcium chloride. An electric air pump propelled the atomized mist into the nozzle. Subsequently, the pressure is adjusted to achieve a flow velocity, resulting in hollow fibers with diameters ranging from 1200 to 1500 μm, as illustrated in Fig. 1c. This technique allows for better control of the gelation rate, preventing excess cross-linking agent accumulation on the printing substrate and, to some extent, mitigating its impact on shape. Eva Mueller et al.^27^ designed a coaxial nozzle with a mixing zone to blend and crosslink functional polymers before extrusion effectively. Introducing the mixing zone improved the uniformity of blending and the stability of fiber formation. However, an excessively long mixing zone or a too-small nozzle diameter may lead to nozzle clogging. Qing Gao^28^ of Zhejiang University added a porous snap to ensure the coaxiality of the coaxial nozzle. The porous snap has a small hole in the center and four large holes at the edge. The central hole is used to fix the position of the inner needle so that the inner needle is coaxial with the outer needle, and the external fluid flows from the tee into the outer needle through the four large holes, as shown in Fig. 1d. However, this method requires high manufacturing and assembly accuracy of the porous snap; otherwise, it is challenging to ensure coaxiality.

Therefore, in this article, we present a semi-flexible coaxial nozzle with a self-centering function, which adopts a structure of nesting a flexible thin-film tube inside a rigid short tube to replace the pure rigid tube. Thus, during the extrusion process, the fluid pressure causes real-time oscillation of the semi-flexible inner needle to ensure the coaxiality of the inner and outer needles, achieving good results with a simple device. We analyzed the influence of external fluid velocity (${v}_{f}$) and inner needle Young's modulus ($E$) on coaxiality error correction performance and self-centering time by finite element simulation. Finally, the self-centering performance of the semi-flexible nozzle is verified by experiments.

## Materials and methods

### Self-centering principle and model construction of semi-flexible coaxial nozzle

**Figure 2:**
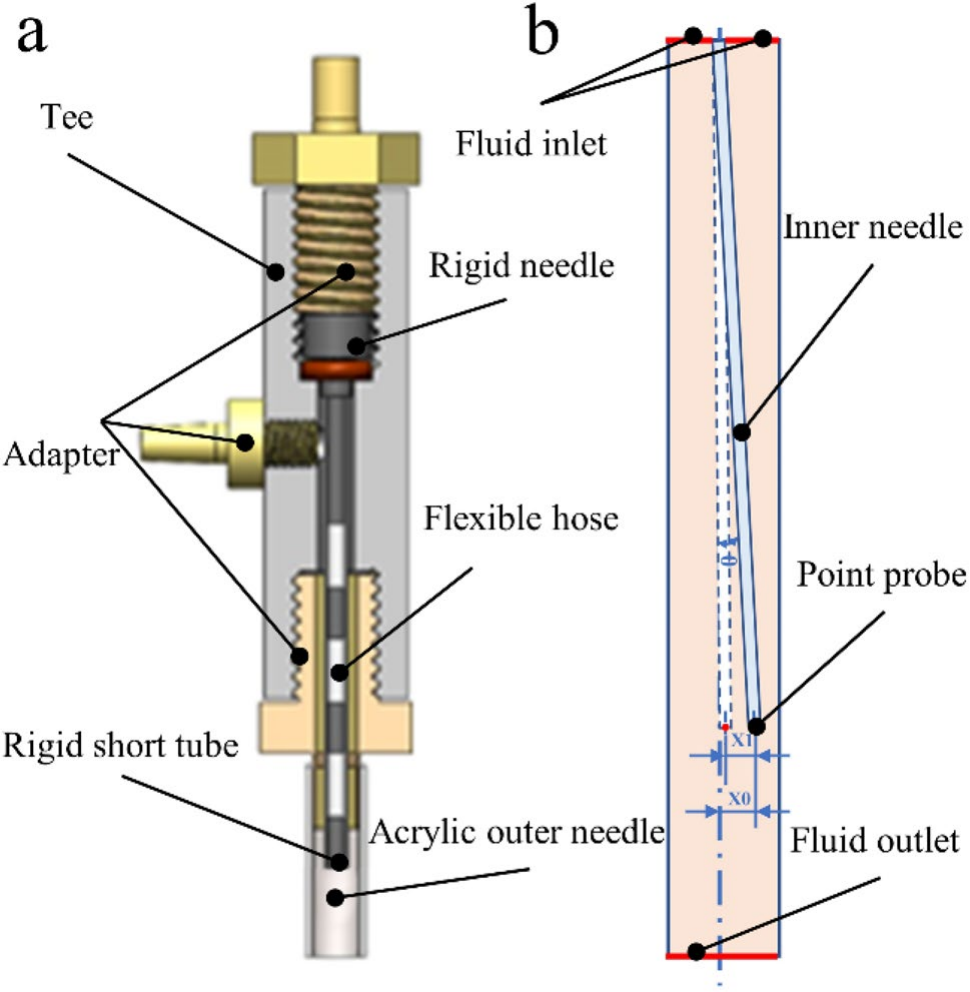
(**a**) Structure of a semi-flexible coaxial nozzle. (**b**) Simplified physical model.

The coaxial nozzle comprises tees, a semi-flexible inner needle, an acrylic outer needle, and an adapter, as shown in Fig. 2a. The core of self-centering is the semi-flexible inner needle, composed of a rigid needle, flexible hose, and several cut-off rigid short tubes. Its self-centering principle is that because the rigid pipe of the inner needle is replaced by a flexible hose and the symmetrical distribution of fluid pressure in the pipe, the inner needle will deform and tend to be centered under the action of the fluid. Moreover, the rigid short tube arranged in blocks not only ensured the tubular structure of the core channel but also maximized the flexibility of the whole structure of the inner needle, that is, the so-called “semi-flexible.” Compared with the fixed structure coaxial nozzle provided in the literature^29^, the combined structure is convenient for changing the extrusion size. Compared with the combined coaxial nozzle^30,31^, the semi-flexible nozzle dramatically reduces component and assembly accuracy requirements. It better balanced the coaxial nozzle's structural complexity and coaxial accuracy.

To facilitate the analysis of the self-centering mechanism of the coaxial nozzle, a simple physical model of coaxiality error is first constructed, as shown in Fig. 2b. The parameters are as follows: the length of the semi-flexible inner needle is 30 mm, and the diameter is 0.5 mm. The angle of deviation from the axis is *θ*, and the distance between the end's central point and the central axis is *X*_0_. The left boundary is the outer fluid inlet of the inner needle, and the right boundary is the fluid outlet. The semi-flexible inner needle is fixed and constrained on the left, and a point probe is set at the center of the right boundary to facilitate subsequent data acquisition. Under the effect of fluid–solid coupling, the semi-flexible inner needle was bent and deformed, and its outlet end generated displacement *X*_1_. Instantaneous coaxiality error was defined to characterize the self-centering performance of the semi-flexible inner needle *e*:

1 $$\begin{array}{c}e={X}_{0}-{X}_{1},\end{array}$$

It is assumed that the flowing liquid is an incompressible Newtonian fluid, the fluid flow is laminar, the density is 1030 kg/m^3^, and the dynamic viscosity is 0.0035 Pas, regardless of gravity. The outer needle is an impenetrable rigid wall; Fluid motion follows the continuity equation and the Navier–Stokes equation^32,33^:

2 $$\begin{array}{c}\rho \frac{\partial u}{\partial t}+\rho \left(u\nabla \right)u+\nabla p-\mu {\nabla }^{2}u=0,\end{array}$$

where:* μ* is dynamic viscosity;* u* is the velocity at moment *t*; *p* is pressure; ∇ is the Hamiltonian differential operator; ρ is the fluid density.

Considering the effect of the flow field on the semi-flexible inner needle, integrating the fluid force on the fluid microelement, and then acting on the solid structure, the following formula can be obtained:

3 $$\begin{array}{c}F\left(t\right)=\int {l}_{d}{\tau }_{f}\cdot {d}_{x},\end{array}$$

where ${\overrightarrow{d}}_{x}$ is the displacement vector in the solid domain; ${\overrightarrow{\tau }}_{f}$ is the stress vector in the fluid domain; *l*_*d*_ is the virtual displacement of solid.

Due to the effect of fluid loading, the semi-flexible inner needle undergoes bending deformation, with the maximum displacement occurring at the far right end. Therefore, the curvature equation of the semi-flexible inner needle axis can be derived, which is an approximate differential equation for the deflection curve of the semi-flexible inner needle:

4 $$\begin{array}{c}\frac{{{d}^{2}X}_{1}}{d{x}^{2}}=\frac{1}{\rho \left(x\right)}=\frac{M\left(x\right)}{E{I}_{z}}.\end{array}$$

*EI*_z_ is the flexural stiffness; *M*(*x*)is the bending moment function.

By integrating the differential equation for the deflection curve of the semi-flexible inner needle twice, the following equation can be obtained:

5 $$\begin{array}{c}{X}_{1}=\iint \frac{M\left(x\right)}{E{I}_{z}}dxdx+Cx+D.\end{array}$$

That is:

6 $$\begin{array}{c}e={X}_{0}-\iint \frac{M\left(x\right)}{E{I}_{z}}dxdx+Cx+D\end{array}$$

where C and D are integral constants, which the boundary conditions of the semi-flexible inner needle can determine.

The object of this study consists of a semi-flexible inner needle and an outer needle flow channel. Using the simulation software (COMSOL Multiphysics 6.0, COMSOL Inc, Stockholm, Sverige) as the computational platform, the coaxiality error model is embedded into the simulation calculation to investigate the relationship between the superficial flow velocity (${v}_{f}$), the stiffness of the inner needle ($E$), the centering time, and the coaxiality error.

### Materials

Sodium alginate (chemical pure CP, relative molecular mass 216.1, Sinopharm Chemical Reagent Co., Ltd). Calcium chloride (analysis pure AR, relative molecular mass 110.8, Sinopharm Chemical Reagent Co., Ltd). Sodium alginate was dissolved in deionized water to prepare an aqueous solution with a concentration of 4%, which was stirred by a magnetic agitator at room temperature for 10 h (rotation speed 120r min^–1^) and used as shell fluid material. Calcium chloride was prepared in deionized water with a concentration of 3% as the core fluid material. Ammonium Violet urate (Tianjin Damao Chemical Reagent Factory) is used to distinguish the core and shell parts for visualization.

### Preparation of semi-flexible coaxial nozzle

**Figure 3:**
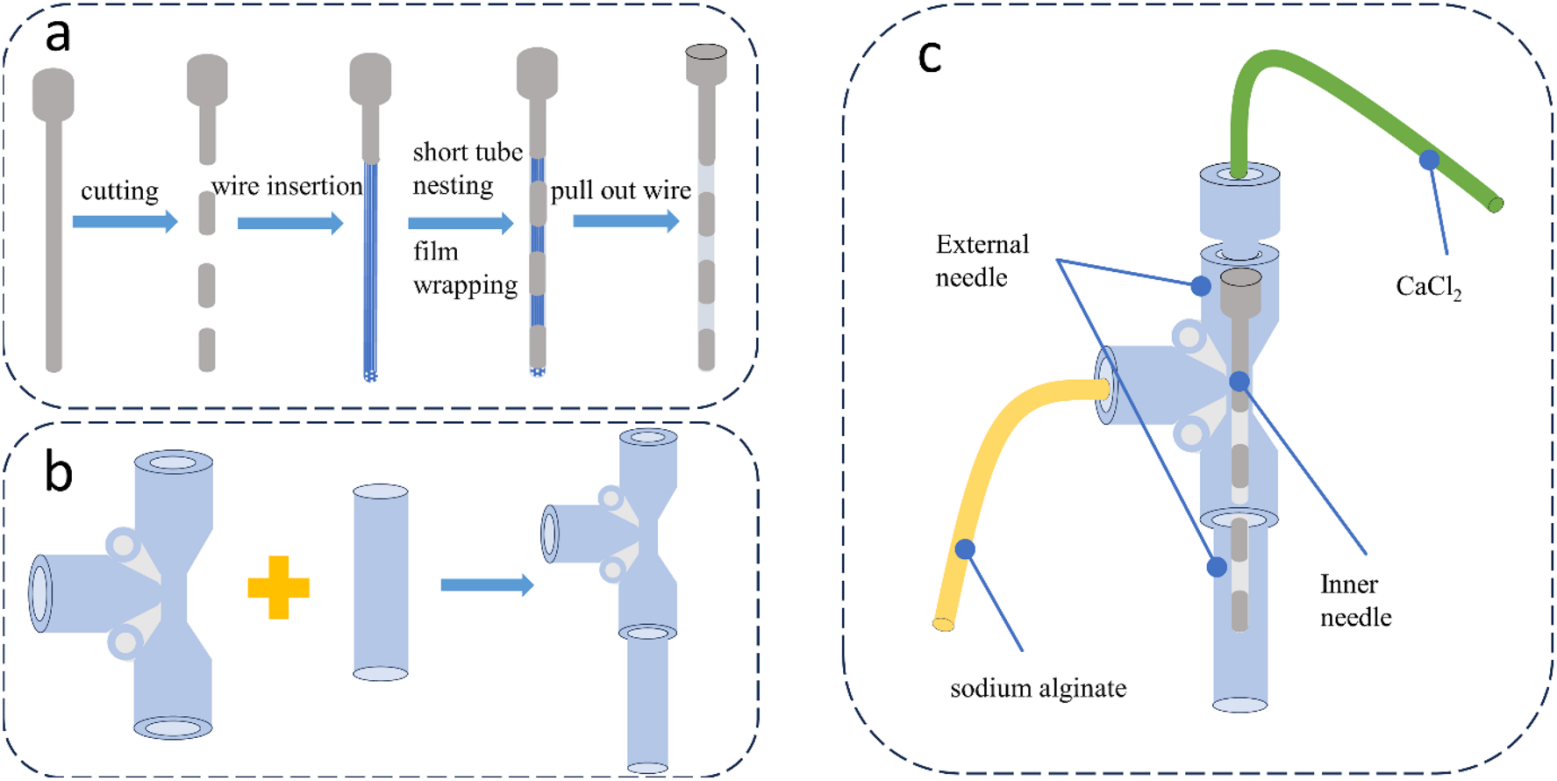
The preparation process of the semi-flexible coaxial nozzle (**a**) Preparation of inner needle (**b**) Assembly of outer needle (**c**) Schematic diagram of semi-flexible coaxial nozzle.

The preparation process of the semi-flexible coaxial nozzle is shown in Fig. 3. First, the rigid needle tube is cut into two parts: the needle head and the short tube. Fine copper wires are filled into the needle head part, and the short tube is evenly arranged in a segmented manner on the fine copper wires. Then, glue is applied to the needle head part, and polyethylene film is tightly wound around it to form a flexible hose. Finally, the fine copper wires are pulled out to obtain a semi-flexible needle head. The semi-flexible inner needle head is tightly connected to the outer needle head to obtain the semi-flexible coaxial nozzle used in this paper.

### Preparation of calcium alginate hollow fibers

**Figure 4:**
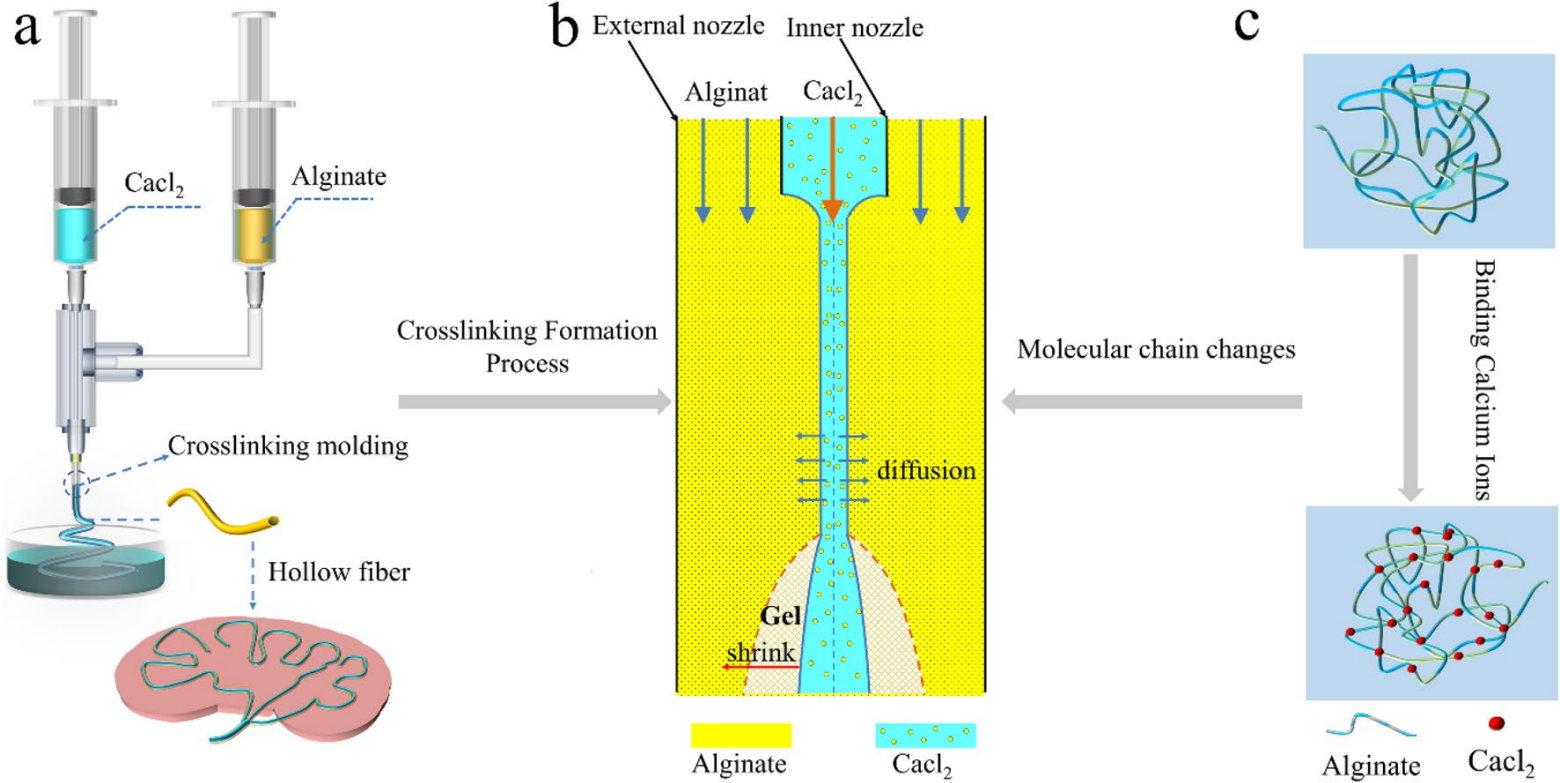
Preparation of calcium alginate hollow fibers (**a**) coaxial extrusion process (**b**) the crosslinking principle of calcium alginate hollow fibers (**c**) changes in molecular chains.

The crosslinking principle of calcium alginate hollow fibers is shown in Fig. 4. We use our homemade semi-flexible coaxial nozzle to coextrude sodium alginate and calcium chloride (the core is filled with calcium chloride solution, and the shell is filled with sodium alginate solution). Under the drive of a motor, the injector is used to extrude the materials. When sodium alginate and calcium chloride come into contact, Ca^2+^ diffuses radially from the inside to the outside. It continuously chelates and solidifies with the alginate molecules in the shell, forming a stable hollow fiber structure. During this process, the calcium chloride solution in the core not only provides the required Ca^2+^ for the cross-linking reaction but also supports the internal space of the fiber, preventing the collapse of the hollow fiber and ensuring that the produced fibers are more stable and uniform. A receiving bath filled with calcium chloride solution is placed below to further cross-link and solidify the gel.

### Detection method

**Figure 5:**
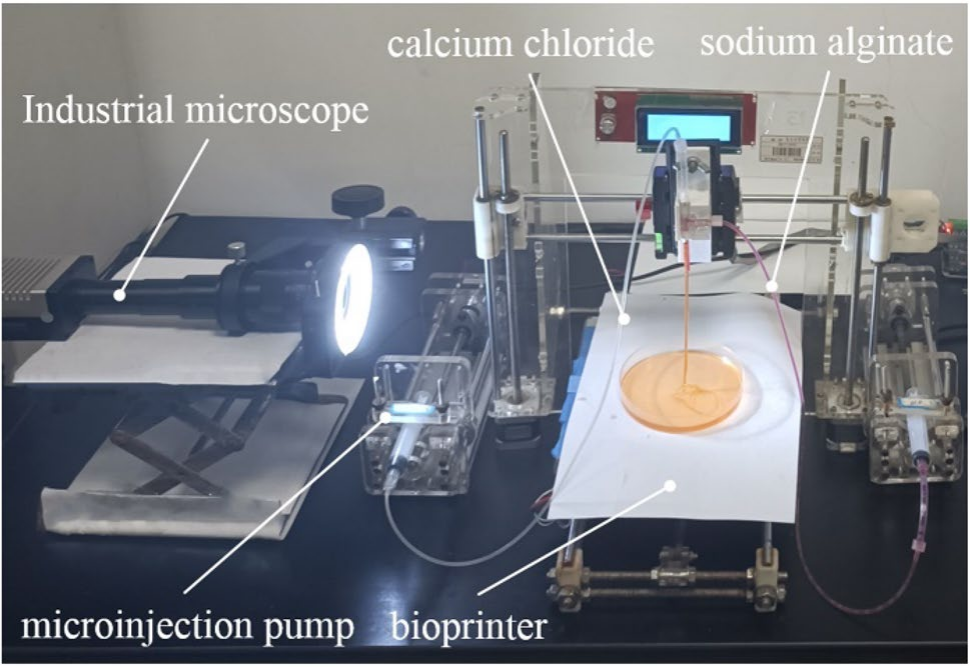
Observation platform for hollow fiber fabrication process.

Observation of the positional changes of the semi-flexible inner needle tip under the influence of coaxial fluid is carried out in real time through the recording function of an industrial microscope. The device is shown in Fig. 5. The hollow fiber is formed by cross-linking between coaxial fluids, the wall thickness of the hollow fiber is measured, and the coaxiality self-correcting performance of the coaxial nozzle is proved indirectly.

In order to maintain consistent flow patterns in two-phase flow, a fixed velocity ratio of 2 is utilized. Table 1 presents the feed combinations of four groups with varying flow rates.

**Table 1:** Combination of constant velocity ratio (m/min).

Material	Combination			
	I	II	III	IV
Sodium alginate	0.03	0.09	0.15	0.21
Calcium chloride	0.015	0.045	0.075	0.105

## Results

### Transient analysis results

**Figure 6:**
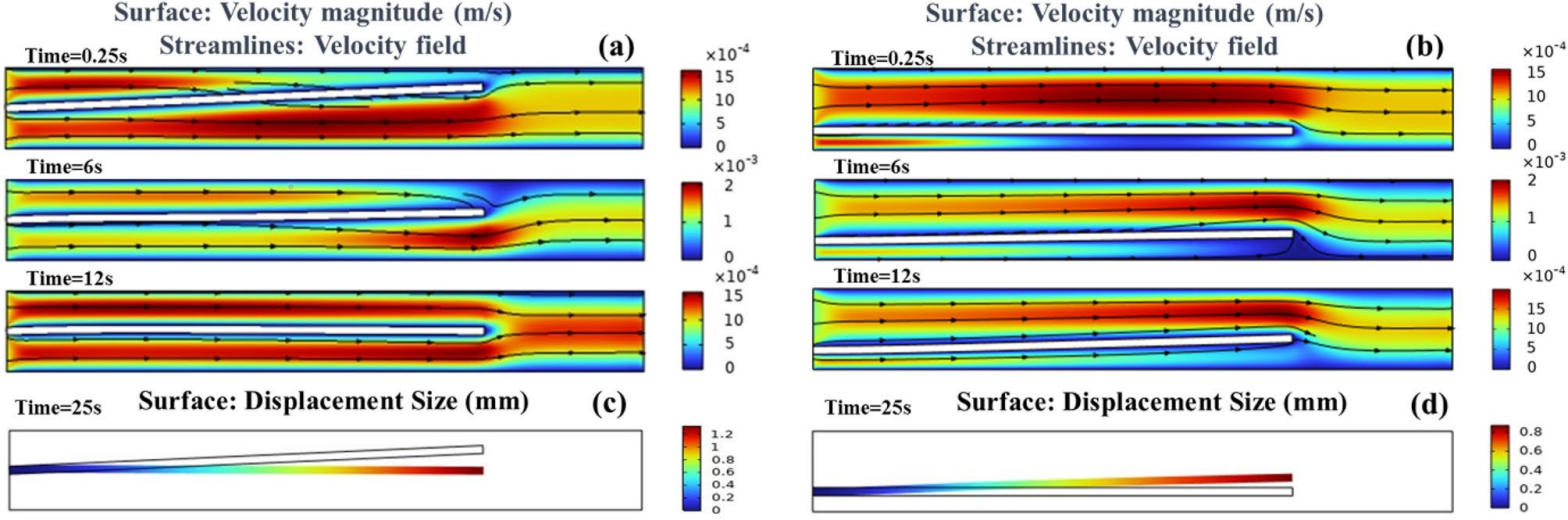
Fluid–solid coupling transient analysis of coaxial nozzle, ((**a**) the distribution of the fluid velocity field at different times when the inner needle inlet is centered, (**b**) the fluid velocity field distribution at different times when the inner needle inlet is offset, (**c**) the inner needle surface displacement diagram when the inner needle inlet is centered, (**d**) the inner needle surface displacement diagram when the inner needle inlet is offset. *v*_*f*_ = 0.015 m/min, inner needle *E* = 0.1 MPa).

The transient analysis results of the coaxial nozzle's internal flow field and the surface displacement of the inner needle are shown in Fig. 6a. It can be observed that the inner needle gradually undergoes bending deformation over time, with its distal end gradually centering. The initial coaxiality deviation of the inner needle leads to a significant difference in the flow velocity (${v}_{f}$) above and below it. The farther the inner needle deviates from the centerline locally, the greater the fluid velocity below and the smaller the fluid velocity above. As time progresses, under the influence of the flow field, the inner needle gradually centers itself, and the ${v}_{f}$ on both sides becomes essentially equal. According to the Bernoulli equation, it is known that regions with higher ${v}_{f}$ have lower pressure. Therefore, the pressure on the upper side of the inner needle is greater than the pressure on the lower side, causing the inner needle to bend downwards until it reaches a centered position, where the pressure difference between the two sides and the resistance to the needle deformation are balanced. Additionally, a comparative experiment was conducted with the initial position of the inner needle deviating from the center, as shown in Fig. 6b. The experiment shows that even in a state of initial positional deviation, the inner needle tends to center gradually under the influence of the fluid.

### Analysis of factors affecting self-centering performance

The influence of Young's modulus ($E$) on the centering time and coaxiality error of the inner needle is shown in .

**Figure 7:**
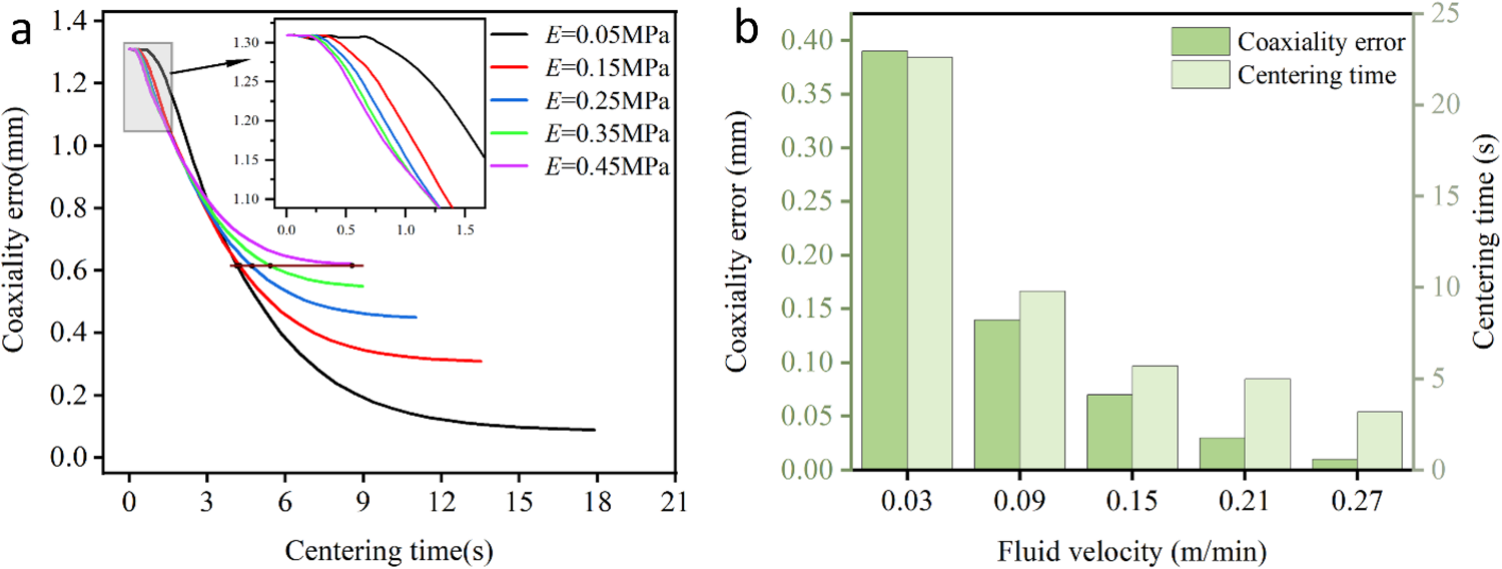
Variation trend of coaxiality error and centering time. (**a**) The influence of $E$ (${v}_{f}$=0.015 m/min). (**b**) The influence of ${v}_{f}$ ($E$=0.05 Mpa).

Figure 7a, the coaxiality error of the inner needle increased with the increase in $E$. Under the action of the fluid, as the centering time increased, the coaxiality error of the inner needle for different $E$ values dynamically reduced. However, the final coaxiality errors of the inner needle differ when it reaches force balance. The larger the $E$ value, the larger the limit of coaxiality error. When the $E$ value of the inner needle is 0.45 MPa, the coaxiality error at force balance is 0.62 mm. When the $E$ value of the inner needle is 0.05 MPa, the coaxiality error decreases to 0.09 mm at force balance.

For the centering time, because the final position of each needle is different, we examined the time required for all inner needles to reach a dynamic coaxiality error of 0.62 mm. It can be observed that the inner needles with larger $E$ values require a longer time, with a maximum of 8.5 s (0.45 MPa) and a minimum of 4.1 s (0.05 MPa). The time the inner needle reaches force balance and tends to center decreases with increasing $E$ values. When $E$ is 0.45 MPa, the centering time at force balance (coaxiality error = 0.62 mm) is 8.5 s. When $E$ is 0.05 MPa, the centering time at force balance (coaxiality error = 0.09 mm) is 17.5 s. There is a noteworthy detail: in the initial stage of the inner needle centering movement (three seconds ago), the larger the $E$, the faster the inner needle is centered. This could be because when the semi-flexible inner needle is acted by the fluid, the front part of the needle is subjected to force first. On the other hand, the softer the semi-flexible inner needle is, the easier it's to deform, so the front part would be centered first, while the end (at the point probe) is almost unchanged. If $E$ is too large and the semi-flexible inner needle is not easy to deform, the overall force would decrease, and the coaxiality error would decrease rapidly. With the continuous flow of the fluid, the rear part of the semi-flexible inner needle is also affected by the liquid. At this time, the softer the material is, the faster the coaxiality error decreases. Therefore, taking coaxiality correction as the first requirement, the softer the material of the inner needle, the better ($E$); while considering the need for centering time, retaining a certain stiffness is necessary.

The variation pattern of the centering time with ${v}_{f}$ for the coaxiality error and the inner needle force balance is shown in Fig. 7b. It can be seen that with the increase of ${v}_{f}$, the coaxiality error and the centering time of the inner needle decreased significantly. When ${v}_{f}$ increased from 0.03 to 0.09 m/min, the coaxiality error of the inner needle decreased from 0.39 to 0.14 mm; meanwhile, the centering time of the inner needle decreased from 22.6 to 9.80 s. When ${v}_{f}$ is increased to 0.27 m/min, the coaxiality error of the inner needle is reduced to 0.01 mm, and the centering time is shortened to 3.2 s. However, when the ${v}_{f}$ continued to increase, the coaxiality error and centering time of the semi-flexible inner needle were almost unchanged. The main reason for the above phenomenon is that with the increase of the inlet ${v}_{f}$, the pressure difference between the upper and lower part of the inner needle increased, resulting in the final coaxiality error and centering time decreased. It can be seen that the semi-flexible inner needle of the same material (the same $E$), the coaxiality error and the centering time of the semi-flexible inner needle can be further reduced by increasing ${v}_{f}$ to a certain extent.

### Self-correcting performance of semi-flexible coaxial nozzle

**Figure 8:**
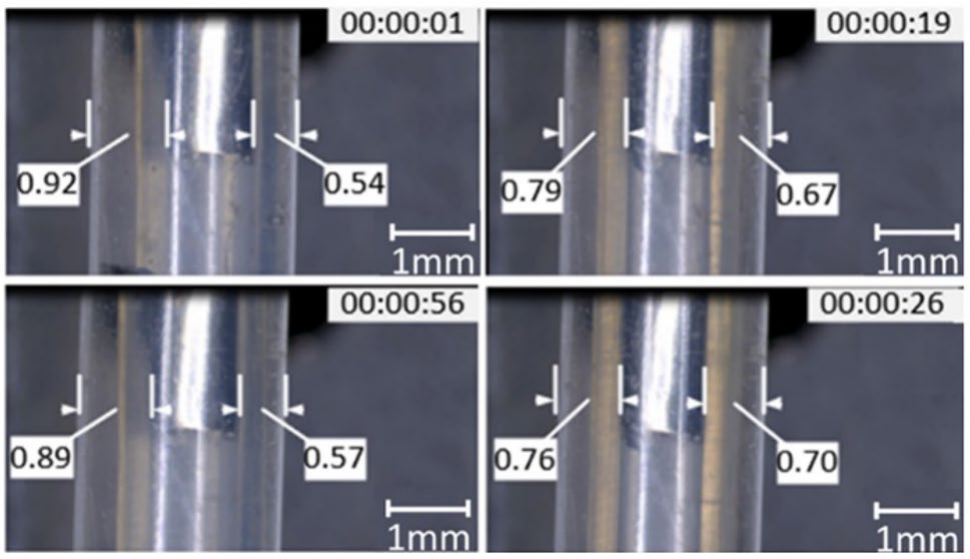
Online monitoring of coaxiality self-correcting effect (0.03 m/min and 0.015 m/min).

Figure 8 shows the position of the needle in the coaxial flow field at different times. Before entering the two-phase flow, the end of the semi-flexible inner needle deviated from the center. With the slow inflow of the two-phase flow liquid, the semi-flexible inner needle is gradually centered below the action of the fluid. The optimal centering effect is reached at about 26 s; at this time, the coaxiality error is 0.06 mm. However, after stopping the fluid flow for some time, the semi-flexible inner needle gradually returned to the original initial position and was still in an eccentric state. The above phenomenon fully proved that the semi-flexible inner needle could realize the function of self-centering under the action of a two-phase flow fluid.

**Figure 9:**
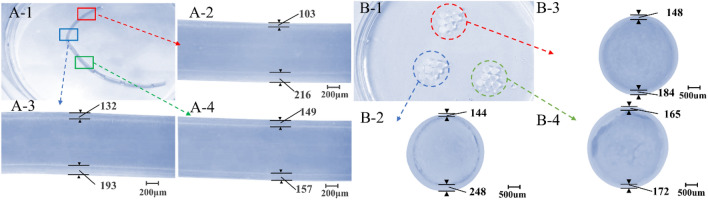
The indirect demonstration of the self-correction performance of coaxiality. (**A**) The variation in the wall thickness of hollow fibers; (**B**) The variation in the wall thickness of gel microspheres).

A section of hollow fiber printed under the above conditions is shown in Fig. 9A-1. In order to facilitate the characterization of hollow performances, some bubbles are injected into the printed fiber. Therefore, the wall thickness structure size of the hollow fiber can be clearly seen. In the initial stage, due to the severe eccentricity of the inner needle, the fiber wall thickness is uneven; the thinnest is 103 μm, and the thickest is 216 μm, as shown in Fig. 9A-2. With the semi-flexible inner needle gradually centered, the wall thickness of the printed hollow fiber gradually became uniform, and the wall thickness (i.e. coaxiality error) difference finally stabilized at about 8 μm, as shown in Fig. 9A-3,A-4. Although the cross-linking shrinkage resulted in the difference between the fiber wall thickness and the layer thickness observed by Sol online, it could also reflect the self-correcting effect of coaxiality. Similarly, experiments related to gel microspheres were conducted, and gel microsphere wall thickness gradually became uniform under the self-correcting performance of the nozzle, as shown in Fig. 9B-1–B-4. The experiments have demonstrated the applicability of the self-correction capability of the semi-flexible coaxial nozzle under various experimental conditions.

**Figure 10:**
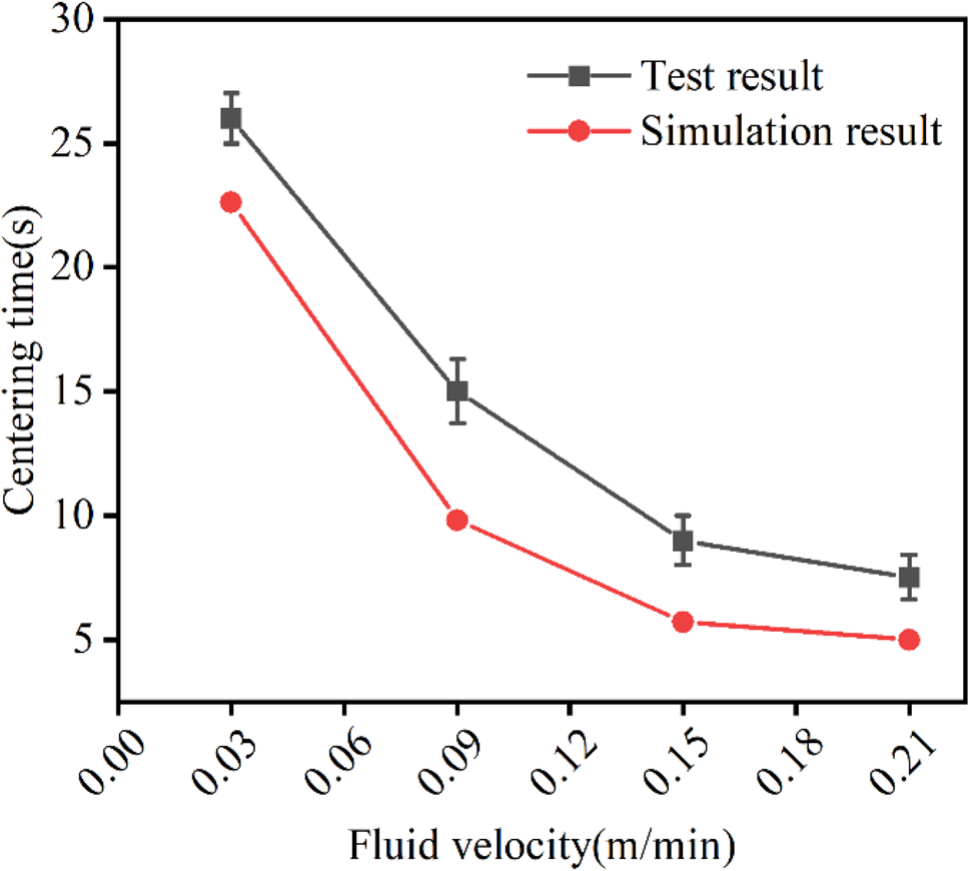
Verification and comparison of centering time.

In this paper, the influence law of ${v}_{f}$ is verified by experiments. It can be seen from Figure 10 that the centering time of the inner needle decreased with the increase of the two-phase flow velocity. When the average flow rate increased to 0.21 m/min, the corresponding centering time decreased from 26 to 7.2 s. The trend of the experimental results is basically consistent with the simulation results, which also proved the effectiveness of the simulation prediction of the test results through the established simulation model. However, there's a certain deviation between the test results and the finite element simulation results, mainly because of the divergence between the simplified structure and material parameters of the simulation model and the actual situation of flexible thin film nested rigid short tubes. In addition, the centering effect of semi-flexible and fully flexible structures is compared, and it is found that the centering time of fully flexible tubes is slightly shorter than that of semi-flexible tubes, while the coaxiality error is almost the same. However, there is a big problem with the fully flexible inner needle. When the viscosity of the liquid supplied by the outer needle is large, it is difficult for the inner needle to support the cylindrical inner cavity, so it is difficult to guarantee the formation of the coaxial flow pattern. In particular, the cross-linked coaxial fluid is more prone to blockage due to uneven cross-linking.

## Discussion

Currently, common coaxial nozzles feature a rigid composite structure, limiting their flexibility and increasing production costs. Tight connections are required to ensure their coaxiality; otherwise, it's difficult to maintain coaxial flow of inner and outer fluids, resulting in significant eccentricity of hollow fibers. This compromises the dimensional and shape accuracy of the produced hollow fibers, failing to meet application requirements. While ensuring the coaxiality of the coaxial nozzle, and considering the flexibility of the nozzle, we propose an innovative semi-flexible coaxial nozzle with self-centering functionality. Based on the symmetrical distribution of fluid pressure inside the channels and the fluid–structure coupling effect, it can correct the deviation of the inner needle during the extrusion process.

**Figure 11:**
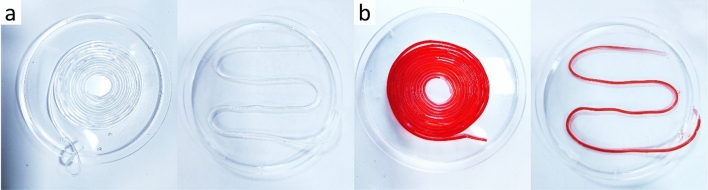
Red ink infused hollow fibers (**a**) before infusion (**b**) after infusion.

Hollow fibers prepared by the semi-flexible coaxial nozzle shown in Fig. 11. When red ink is infused into the hollow channels, complete passage through the channels can be observed. The printed alginate hollow fibers are continuous, with uniform diameters, and possess intact structure.

**Figure 12:**
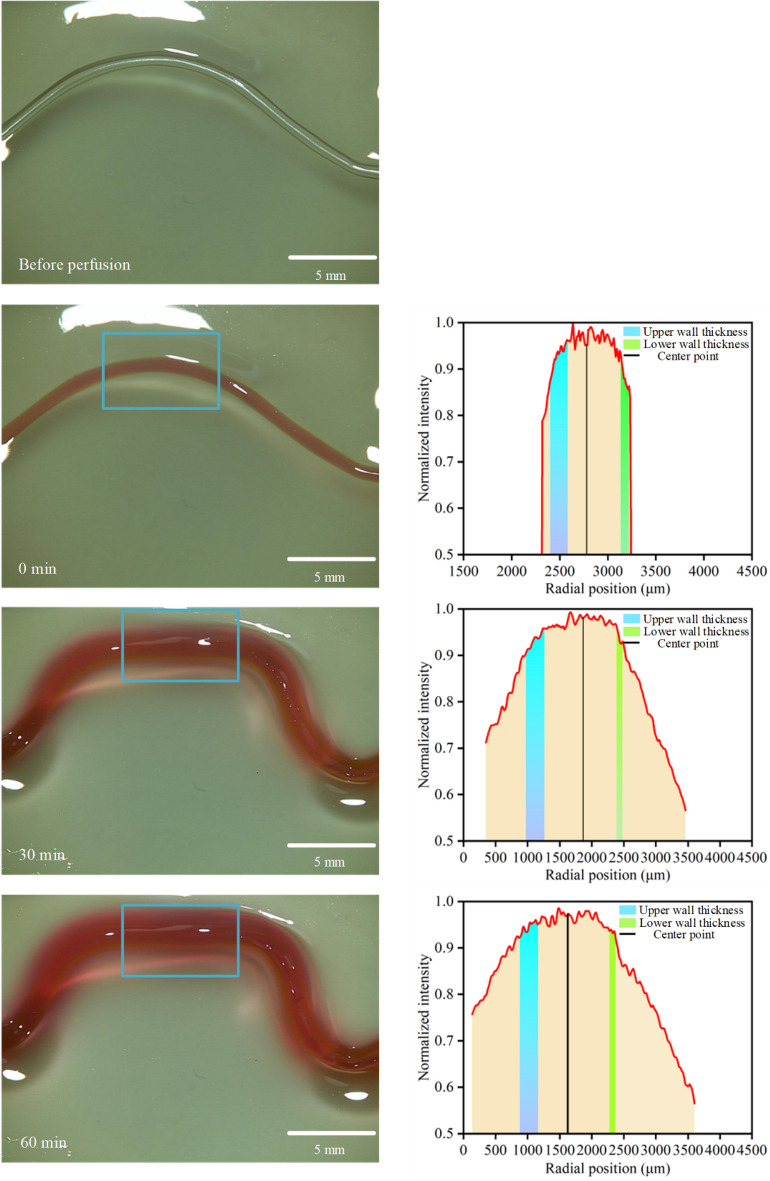
Permeation process of hollow fibers.

Due to its excellent biocompatibility, calcium alginate hollow fibers can be used to replace or assist autologous blood vessels. Their permeability needs to meet the requirements of cellular metabolism and nutrient transport. The consistency of fiber wall thickness is crucial for their permeability. Uniform wall thickness ensures even penetration of substances at different positions; if the thickness is uneven, the permeability may vary at different locations. Thicker sections may restrict substance penetration, while thinner sections exhibit higher permeability. Figure 12 illustrates the permeation process of calcium alginate hollow fibers with uneven wall thickness. With increasing time, the stained solution gradually diffuses and permeates to both sides. The upper side, with thicker wall thickness, shows limited permeability, whereas the lower side, with thinner wall thickness, exhibits significantly higher permeability than the upper side. During the permeation process, the fibers bend into a quasi-letter shape due to the difference in wall thickness between the upper and lower sides. Thinner wall thickness on the lower side causes greater swelling upon water absorption, leading to overall bending of the fibers downward. Therefore, to ensure consistent permeability of the fibers, it is essential to maintain uniform wall thickness, highlighting the importance of nozzle concentricity in fiber formation quality.

In terms of molding precision, Cornock^34^ used alginate-poly(ε-caprolactone) core-sheath fiber formed by their coaxial melt extrusion printing device, and the core offset is 60 μm; Ibrahim T Ozbolat^24^ utilized a hollow filament form of printable microfluidic channels to mold hollow fibers, achieving a wall thickness deviation of 26 μm; Qing Gao^28^ printed and molded hollow fibers with a stable wall thickness deviation of around 28 μm; Yahui Zhang^35^ achieved a wall thickness difference of approximately 42 μm in the 3D printing of hollow fibers; Rana Attalla^36^ obtained a fiber wall thickness difference of 25 μm using a custom PDMS microfluidic nozzle; Cui di Li^37^ produced hollow fibers with a wall thickness difference of 20 μm. Yin Yu^38^ achieved a wall thickness deviation of approximately 23 μm with printed hollow fibers. Compared to the fiber precision obtained with the devices above, our semi-flexible self-centering coaxial nozzle printing system for molding hollow fibers can maintain a wall thickness deviation of 8 μm, achieving higher accuracy.

Regarding part accuracy and assembly accuracy, the coaxial nozzle's part accuracy and assembly accuracy influence its coaxiality error. Ibrahim T Ozbolat et al.^24^ used stainless steel fixtures made by fine milling to align the two tubes to ensure their coaxiality; Buu Minh Tran^4^ made triple-flow microfluidic devices through mold; Qing Gao^28^ used porous clasps to ensure the coaxiality of coaxial nozzles; Alessandri^39^ and S. Cem Millik^40^ utilized a digital light processing 3D printer to fabricate microfluidic coaxial nozzles. Jin et al.^41^ manufactured a three-layer stainless steel nozzle, assembled through bolts, and fine-tuned the alignment of the three channels coaxially under a microscope. Jiaxing Gong et al.^42^ used epoxy resin to concentrically fix the inner and outer needles to ensure the coaxiality of the nozzle. Di Wang et al.^43^ designed a BNS bioprinting nozzle system and utilized a stereolithography printer to 3D print the components of the nozzle. Subsequently, the nozzle was assembled using screws and bolts.

For the above-mentioned coaxial nozzles, these parts try to keep the coaxiality error of the nozzle to a minimum by maintaining high precision in the manufacturing and assembly process. However, our coaxial nozzle, due to its unique semi-flexible structural design, can automatically make fine adjustments utilizing fluid during the extrusion process, which can make up for the lack of part accuracy and assembly accuracy to a large extent to achieve higher coaxiality accuracy. This design can effectively reduce the requirements for parts and assembly accuracy and improve the reliability and stability of the product. Generally speaking, the semi-flexible coaxial nozzle is a kind of nozzle with highly adaptive performance, which can not only reduce the precision requirements for parts and assemblies during the molding process but also usually work in a non-ideal manufacturing environment. And ensure the molding quality of the fiber.

Although the nozzle can stabilize the wall thickness difference of the formed hollow fiber at about 8 μm, there is still a certain gap from the ideal state of the inner needle being perfectly centered. Further research and exploration are still required. Firstly, in terms of flexible film materials, more flexible film can be tested and used to reduce coaxiality errors further. Secondly, using computational fluid dynamics simulations and optimization methods, detailed numerical simulations and analyses of the internal flow in the nozzle can be performed to identify the sources of flow non-uniformity and propose corresponding improvement strategies. The uniformity of fiber wall thickness is improved by optimizing nozzle structure, flow channel layout, and fluid parameters. The effects of different materials' rheological properties and processing conditions on fiber wall thickness can be further studied to optimize the selection of materials and the setting of processing parameters. This study provides a novel geometric control method for the printing and shaping of hollow fibers, offering valuable reference and solutions for addressing issues such as uneven wall thickness caused by coaxial errors during the extrusion process.

## Conclusions

This paper provided an innovative semi-flexible self-centering coaxial nozzle, and its self-centering performance and influencing factors are analyzed and verified by finite element simulation and experiments.

1. The self-centering mathematical model of the coaxial nozzle is established based on the principle of hydrodynamics and fluid–solid coupling.
2. The coaxiality error of the inner and outer needles of the coaxial nozzle increases with the increase of Young's modulus $E$ of the inner needle. It decreases with the increase of the fluid velocity, and the centering time of the force balance of the inner needle decreases with the increase of Young’s modulus $E$ and the fluid velocity. However, during the initial stage of the centering motion of the inner needle (three seconds ago), the coaxiality error and centering time decrease with an increase in the inner needle's Young’s modulus $E$.
3. The semi-flexible coaxial nozzle has a remarkable self-centering effect, which could dynamically reduce the original coaxiality error and the precision requirements for coaxial nozzle parts and assembly.

## Data Availability

All data used in the current study can be found in the article.
